# Dietary Supplementation with Probiotics Improves Hematopoiesis in Malnourished Mice

**DOI:** 10.1371/journal.pone.0031171

**Published:** 2012-02-08

**Authors:** Susana Salva, María Cecilia Merino, Graciela Agüero, Adriana Gruppi, Susana Alvarez

**Affiliations:** 1 Reference Centre for Lactobacilli (CERELA-CONICET), Tucuman, Argentina; 2 Department of Clinical Biochemistry, School of Chemical Science, National University of Cordoba, Cordoba, Argentina; 3 Clinical Biochemistry, School of Biochemistry Chemistry and Pharmacy, National University of Tucuman, Tucuman, Argentina; Emory University School of Medicine, United States of America

## Abstract

**Background:**

*Lactobacillus rhamnosus* CRL1505 (Lr) administered during the repletion of immunocompromised-malnourished mice improves the resistance against intestinal and respiratory infections. This effect is associated with an increase in the number and functionality of immune cells, indicating that Lr could have some influence on myeloid and lymphoid cell production and maturation.

**Objective:**

This study analyzed the extent of the damage caused by malnutrition on myeloid and lymphoid cell development in the spleen and bone marrow (BM). We also evaluated the impact of immunobiotics on the recovery of hematopoiesis affected in malnourished mice.

**Methods:**

Protein malnourished mice were fed on a balanced conventional diet for 7 or 14 consecutive d with or without supplemental Lr or fermented goat's milk (FGM). Malnourished mice and well-nourished mice were used as controls. Histological and flow cytometry studies were carried out in BM and spleen to study myeloid and lymphoid cells.

**Results:**

Malnutrition induced quantitative alterations in spleen B and T cells; however, no alteration was observed in the ability of splenic B cells to produce immunoglobulins after challenge with LPS or CpG. The analysis of BM B cell subsets based on B220, CD24, IgM and IgD expression showed that malnutrition affected B cell development. In addition, BM myeloid cells decreased in malnourished mice. On the contrary, protein deprivation increased BM T cell number. These alterations were reverted with Lr or FGM repletion treatments since normal numbers of BM myeloid, T and B cells were observed in these groups.

**Conclusions:**

Protein malnutrition significantly alters B cell development in BM. The treatment of malnourished mice with *L. rhamnosus* CRL1505 was able to induce a recovery of B cells that would explain its ability to increase immunity against infections. This work highlights the possibility of using immunobiotics to accelerate the recovery of lymphopoyesis in immunocompromised-malnourished hosts.

## Introduction

Protein malnutrition is the most common cause of acquired immunodeficiency in the world. The relationship between nutritional status and immunity of the host, where malnutrition increases susceptibility to infections and the infections deteriorate the nutritional status [1], [2], could lead to death. In synergy with infection, malnutrition contributes to 56% of all childhood deaths worldwide [3]. Adequate and prompt correction of nutritional status is important to reduce morbidity and mortality from infectious diseases associated with immunodeficiency caused by malnutrition [4]–[6]. However, a simple intervention, such as adequate intake of nutrients in the presence of recurrent infections, is not enough to reverse this cycle because infections themselves cause a critical loss of proteins, energy, vitamins and mineral deposits of the body [3].

One of the reasons why the malnourished are susceptible to infections is that malnutrition alters both innate and adaptive immune response [7]–[10]. Multiple abnormalities of immune response were described as consequences of malnutrition: decrease of phagocytosis and the mobilization of inflammatory cells to the site of infection [11], reduction of the ability to release cytokines by mononuclear phagocytes, decrease of T cell number, and atrophy of the lymphoid tissue [8], [9]. It has been shown that malnutrition affects the tissues that have a high turnover rate and cell proliferation as hematopoietic tissue and induces an impairment of blood cell production, leading to hypoplasia and structural changes of bone marrow (BM) [12]–[14].

Probiotics are defined as live microorganisms that, when administered in adequate amounts, confer a health benefit to the host [15]. Clancy [16] suggested the term “immunobiotic” to identify a bacterium that promotes health targeting on the mucosal immune system. Most probiotic organisms are lactic acid bacteria (LAB), which induce a long list of health benefits [17], [18]. In this sense, by using an experimental model of pneumococcal infection in malnourished (MN) mice, we showed that the administration of LAB during the repletion of MN mice was associated with the improvement of the number and functionality of the immune cells [11], [14], [19]. Moreover, we showed that the *Lactobacillus rhamnosus* CRL1505 administration was able to improve the immunity in experimental models of intestinal and respiratory infections in immunocompetent mice [20]. More recently, we developed and characterized fermented goat's milk containing *L. rhamnosus* CRL1505 and we showed, in a model of MN mice, that the final dairy product preserves the immunomodulatory properties of the strain [21]. Cytochemical studies in blood and BM leukocytes suggest that the effect of LAB in the recovery of MN mice immunity could be related to their influence in the production and maturation of myeloid and lymphoid cells [14]. In the present work, we performed studies to analyze the extent of the damage induced by malnutrition on B cell development in the spleen and BM, extending the study to other myeloid and lymphoid cells. In addition, we evaluated the impact of immunobiotics on the recovery of hematopoiesis affected in immunocompromised MN mice.

## Results

### Protein malnutrition decreased splenic B cell number without affecting their ability to produce immunoglobulins

**Figure 1:**
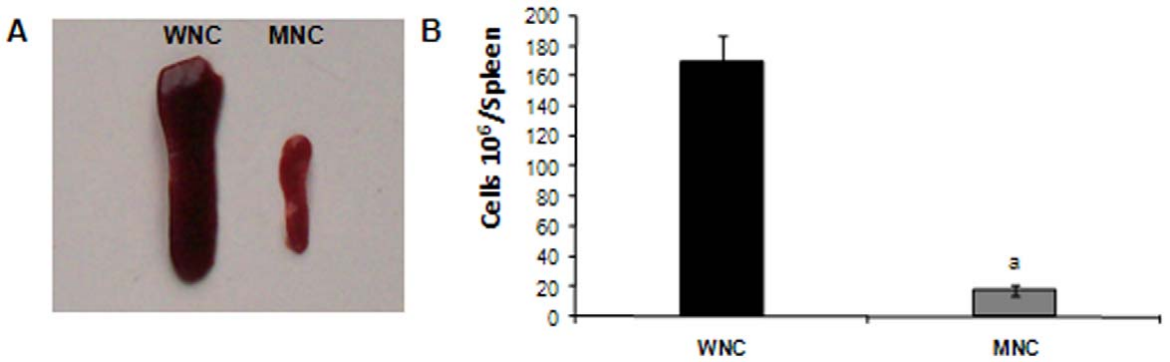
A. Spleens photography obtained from well-nourished (WNC) and malnourished (MNC) mice. B. Total spleen cell number from WNC and MNC mice. The weight of the spleens of WNC and MNC was 178±5 mg and 84±6 mg respectively.

**Figure 2:**
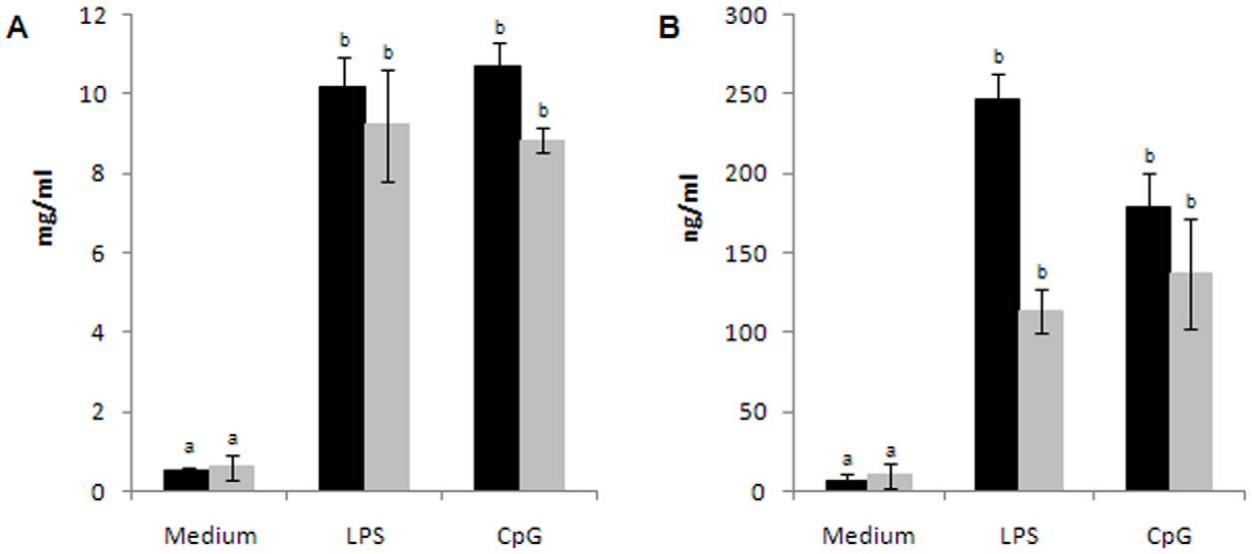
Spleen cells from well-nourished control (WNC) (black bars) and malnourished control (MNC) (grey bars) mice were cultured with medium, LPS or CpG. Total concentration of IgM (A) or IgG (B) in culture supernatants was determined by ELISA. ^a,b^ Means in a bar with different letters (a>b) were significantly different (p<0.01).

Based on the fact that nutritional deprivation affects a specific antibody response [22]–[24], induces atrophy of lymphoid tissues and decreases the number of circulating T and B cells [7], [25], [26], we first studied splenic B cell number in our experimental model. As previously reported [7], [25], [26], we observed that a protein-free diet for 21 days significantly reduced the size of the spleen and the number of total splenic cells (Figure 1A, B). Additionally, the absolute number of mature (B220^+^IgM^+^) and immature (B220^low^CD24^high^) splenic B cells as well as T cell (CD3^+^CD4^+^ and CD3^+^CD4^−^) number were significantly decreased in MN control (MNC) mice in comparison to well-nourished control (WNC) mice (Table 1). Despite the strong reduction in the total B cell number, the proportion of B cells in the spleen of MNC and WNC mice was similar (28,22±0,49% and 26,40±3,67%, respectively). Accordingly, IgM and IgG production by splenic B cells from MNC and WNC mice stimulated with LPS or CpG was similar, suggesting that protein deprivation affects B cell number but not their ability to produce Igs (Figure 2A, B).

**Table 1:** Spleen lymphocytes counts (10^6^ cells/mice).

	WNC	MNC
B220^+^IgM^+^	34,2±5,8^a^	3,1±1,9^b^
B220^low^CD24^high^	6,60±1,7^a^	0,77±0,4^b^
CD3^+^CD4^+^	31,3±6,6^a^	4,6±0,8^b^
CD3^+^CD4^−^	12,5±2,2^a^	0,7±0,1^b^

WNC: Well-nourished control, MNC: malnourished control.

a,b Mean values within a line with unlike superscript letters were significantly different (p<0.05).

### Malnutrition induced a significant reduction in BM cell compartments which were recovered after repletion diet supplemented with probiotics

**Figure 3:**
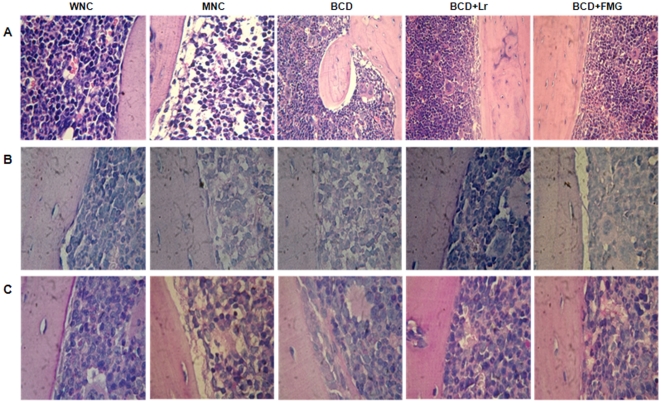
Histological examination of BM architecture from mice well-nourished control (WNC) mice, malnourished control (MNC) mice, and malnourished mice replete for 7 days with a balanced conventional diet (BCD) with supplemental *Lactobacillus rhamnosus* (BCD+Lr) or fermented goat milk (BCD+FGM). The femur was removed; the BM fixed in paraformaldehyde, decalcified in formic acid and sodium citrate, and stained with (A) Hematoxylin and eosin, (B) Alcian Blue or (C) Periodic Acid Schiff. Light micrographs, original magnification ×400.

Considering that the BM is the source of B cells, we continued our study by analyzing the cellularity and histological changes of BM of mice under protein deprivation, particularly on B cell precursors. We observed that malnutrition induces a significant decrease in the number of total BM cells (p<0.001) (Table 2, Figure 3). Through cytological studies we observed that the granulocyte and erythroblast counts in BM of the MNC mice were significantly lower than those found in WNC mice (p<0.01) (Table 2). The groups that received *L. rhamnosus* (Lr) or fermented goats' milk (FGM) normalized the total number of BM cells at day 14 of treatment, while the remaining treatments led to a significant increase in BM cellularity over the MNC mice without reaching normal values. All the repletion treatments normalized granulocyte counts and increased the myelocyte numbers after 7 days of treatment. The administration of a balanced conventional diet (BCD) with Lr (BCD+Lr) and BCD+FGM induced an absolute increase of mitotic pool cells (promyelocytes and blasts) at day 7 of treatment (Table 2). The addition of *L. rhamnosus* or FGM to the repletion diet reduced the time needed for normalization of erythroblast counts at day 7, while for BCD,14 days were required (Table 2).

**Table 2:** BM cell counts (10^6^ cell/femur).

Groups	WNC	MNC	BCD 7d	BCD 14d	BCD+Lr 7d	BCD+Lr 14d	BCD+FGM 7d	BCD+FGM 14d
BM total cells	42.1±2.5^c^	17.9±2.0^a^	31.8±2.9^b^	34.6±2.7^b^	29.4±2.5^b^	41.4±2.0^c^	31.5±2.9^b^	42.2±2.6^c^
Granulocyte cells	16.5±1.5^b^	10.7±1.1^c^	22.6±0.7^a^	9.5±0.6^c^	17.3±1.1^b^	13.1±0.7^b^	19.9±0.7^b^	12.2±0.5^b^
Mitotic pool cells	1.0±0.1	0.3±0.07	5.9±0.1	2.9±0.2	8.1±0.4	6.1±0.2	7.1±0.3	4.7±0.2
Post mitotic pool cells	15.5±1.3	10.4±1.1	16.7±0.5	6.6±0.4	9.2±0.6	7.0±0.5	12.7±0.3	7.4±0.3
Eritroblast cells	19.1±2.3^a^	5.4±0.9^b^	5.3±1.9^b^	20.7±2.4^a^	6.1±1.5^b^	22.2±3.3^a^	7.3±1.7^b^	23.2±2.3^a^

BCD: balanced conventional diet, BCD+Lr: BCD with supplemental *Lactobacillus rhamnosus*, BCD+FGM: BCD with supplemental fermented goat milk, WNC: well-nourished control mice, MNC: malnourished control mice.

Mean SD (n = 6 mice/group) are shown.

a,b,c Mean values within a line with unlike superscript letters were significantly different (p<0.05).

According to cell counts, histological studies revealed that MNC mice had a decrease in the marrow hematopoietic space with a diminution of the BM area occupied by cells in comparison to WNC mice (Figure 3A). Despite the depletion of the erythroid and myeloid compartments, malnutrition is not affecting the megakaryocytic compartment (data not shown). The sinuses from MNC mice appeared to be lengthy and the interstitial areas observed were occupied by a granular acidophilic material associated with local hemorrhage. These regions were Alcian Blue and Periodic Acid Schiff positive (Figure 3B, C), suggesting an increase in the amount of extracellular matrix. The repletion with BCD+Lr or BCD+FGM led to the recovery of the BM hematopoietic space accompanied by the normalization of cellular compartments (Figure 3A). The sinuses were occupied by mature elements of different cell populations. In addition, the endosteal cells retained the architectural rearrangement. The BM of the BCD group generally presented the same characteristics of MNC mice; they only showed a little recovery of hematopoietic spaces, which failed to achieve normality (Figure 3A).

### Protein deprivation affected B cell development in BM which was reverted by supplemented repletion diet

To investigate the effect of nutritional deprivation on the BM lymphocyte compartment we analyzed, by flow cytometry, the lymphocyte number in the BM of MNC mice in comparison with WNC mice. We observed that protein deprivation reduced the total lymphocyte number (Table 3). Repletion with *L. rhamnosus* or with FGM increases the total number of lymphocytes after 7 days of treatment, but the best recovery of the lymphocyte count was observed after 14 days of treatment.

**Table 3:** BM lymphocytes and B cell counts (10^6^cells/femur).

Groups	Lymphocytes	B220^+^ cells
WNC	12.3±1.5^a^	9.11±1.69^a^
MNC	3.6±1.5^d^	2.07±1.04^c^
7d BCD	6.9±1.2^c^	5.65±1.11^b^
7d BCD+Lr	7.4±1.9^c^	4.90±1.32^b^
7d BCD+FGM	8.6±2.8^c^	6.32±2.33^b^
14d BCD	9,14±1,5^b^	8.10±1.68^b^
14d BCD+Lr	15.7±4.6^a^	10.40±4.18^a^
14d BCD+FGM	14.1±3.8^a^	10.35±4.09^a^

BCD: balanced conventional diet, BCD+Lr: BCD with supplemental *Lactobacillus rhamnosus*, BCD+FGM: BCD with supplemental fermented goat milk, WNC: well-nourished control, MNC: malnourished control.

The percentage of lymphocytes obtained from the FSC vs SSC analysis and B220+ cells obtained from B220 histograms were expressed in absolute numbers.

a,b,c,d Mean values within a column with unlike superscript letters were significantly different (p<0.05).

**Figure 4:**
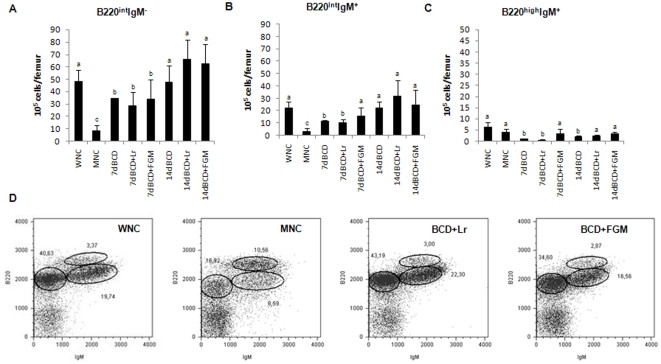
BM cells from malnourished mice replete for 7 days with a balanced conventional diet (BCD) with supplemental *Lactobacillus rhamnosus* (BCD+Lr) or fermented goat milk (BCD+FGM) were stained with fluorochrome labeled anti-mouse B220 and anti-mouse IgM antibodies and analyzed by flow cytometry. Well-nourished control (WNC) and malnourished control (MNC) mice were used as controls. A. Number of B220^int^IgM^−^ cells. B. Number of B220^int^IgM^+^ cells. C. Number of B220^high^IgM^+^ cells is reported. ^a,b,c^Mean values within a column with unlike superscript letters were significantly different (p<0.05). D. Representative plots of B220 vs IgM expression, gated on lymphocytes, are depicted. Results are representative of two independent experiments, n = 6 samples per group.

FACS analysis revealed that the number of B220-positive cells (the whole B-cell compartment) decreased in the BM of MNC mice (Table 3). In parallel with the total B cell decrease, the proportion of B lineage subsets was markedly altered in the BM of fasted mice (Figure 4). Based on B220 and IgM expression, we observed that pro-B/pre-B (B220^interm^IgM^neg^) and immature B cell (B220^interm^IgM^+^) numbers decreased (Figure 4) in MNC mice in comparison with WNC mice. The reduction in immature B cells was accompanied by an increase in the percentage of mature B cells (B220^high^IgM^+^) but not by changes in the number of mature B cells (Figure 4C). These observations suggest that nutritional deprivation leads to the alteration of B-cell development in the BM. The alteration of B lineage cells in the BM of fasted mice was prevented by repletion treatments. The repletion treatment supplemented with the probiotic LAB or with FGM normalized the B cell population after 14 days of treatment (Table 3). Interestingly, the diet supplemented with FGM was the only one that normalized the absolute number of immature B220 cells after 7 days of treatment; the other supplemental diets normalized this population after 14 days of renutrition (Figure 4). Mature B cells were normalized after 14 days of treatment, independently of the diets used. Similar results were obtained when BM B cell subsets were analyzed based on B220, CD24 and IgD expressions (Table S1).

**Figure 5:**
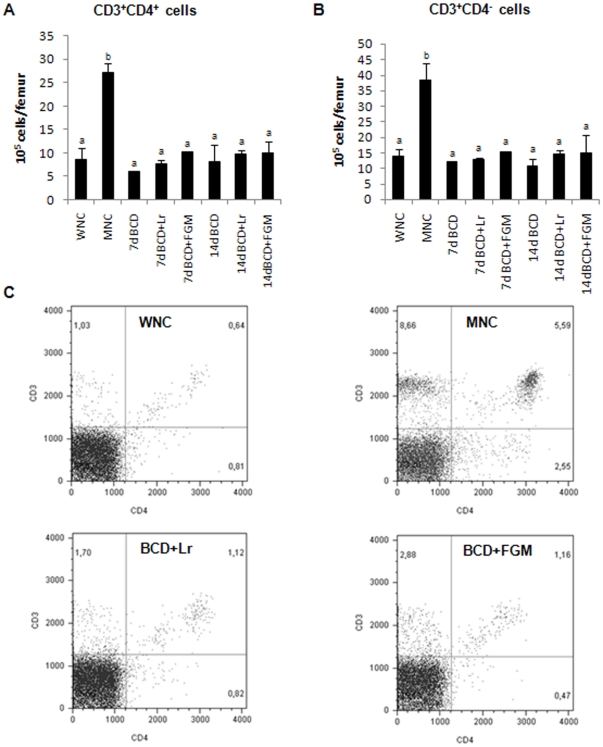
BM cells from malnourished mice replete for 7 days with a balanced conventional diet (BCD) with supplemental *Lactobacillus rhamnosus* (BCD+Lr) or fermented goat milk (BCD+FGM) were stained with fluorochrome labeled anti-mouse CD3 and anti-mouse CD4 antibodies and analyzed by flow cytometry. Well-nourished control (WNC) and malnourished control (MNC) mice were used as controls. A. Number of CD3^+^CD4^+^ cells. B. Number of CD3^+^CD4^−^ cells is reported. ^a,b^Mean values within a column with unlike superscript letters were significantly different (p<0.05). C. Representative plots of CD3 vs CD4 expression, gated on lymphocytes, are depicted. Results are representative of two independent experiments, n = 6 samples per group.

Interestingly, the percentage and number of CD3^+^CD4^+^T cells and CD3^+^CD4^−^ cells increased as a consequence of nutritional deprivation (p<0.05), but they reached the control value after 7 days of repletion in all the treatments tested (Figure 5).

### Protein malnutrition decreased BM myeloid cells but increased T cell number

**Figure 6:**
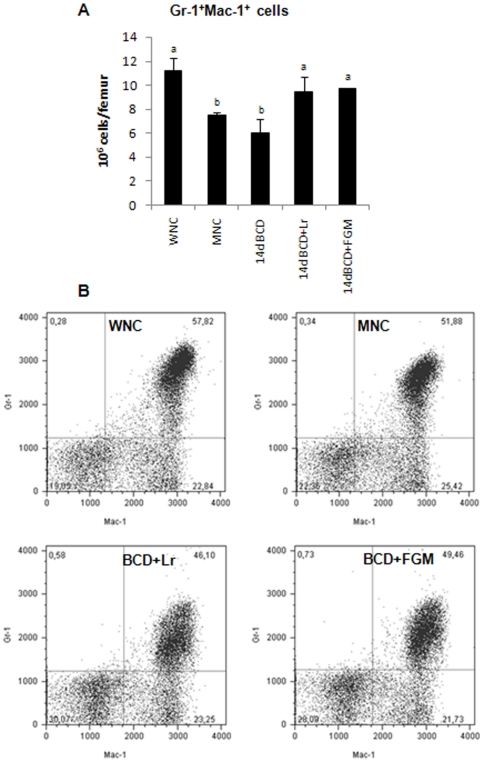
BM cells from malnourished mice replete for 7 days with a balanced conventional diet (BCD) with supplemental *Lactobacillus rhamnosus* (BCD+Lr) or fermented goat milk (BCD+FGM) were stained with fluorochrome labeled anti-mouse Gr-1 and anti-mouse Mac-1 antibodies and analyzed by flow cytometry. Well-nourished control (WNC) and malnourished control (MNC) mice were used as controls. A. Number of Gr-1^+^Mac-1^+^ cells is reported. ^a,b^Mean values within a column with unlike superscript letters were significantly different (p<0.05). B. Representative plots of Gr-1 vs Mac-1 gated on myeloid cells are shown. Results are representative of two independent experiments, n = 6 samples per group.

Despite the fact that there were no differences observed in the percentage of granulocytes (Gr-1^+^Mac-1^+^) between WNC and MNC mice (Figure 6), malnutrition induced a decrease in the total number of granulocytes in BM (Figure 6A). The administration of a repletion diet with *L. rhamnosus* or with FGM induced the recovery of normal values at 14 days of treatment (Figure 6A).

## Discussion

The increase in antibiotic resistance and the need for new and improved strategies to tackle infectious diseases have led to an examination of the therapeutic potential of commensal induced modulation of the mucosal immune response. Consequently, it has been discovered that certain LAB do have protective effects against bacterial and viral infections in the gastrointestinal and respiratory systems [18], [27], [28]. Nowadays, immunobiotics are an attractive and safe way to regulate and enhance immune function, particularly in immunodeficiencies [17], [18], [29]. Here, we reported that protein malnutrition induces a significant reduction in BM cell compartments, which is reflected in a decrease in mature B cells and which was reverted by diets supplemented with probiotics.

It has been reported that an inadequate protein diet diminishes the immune response and increases the susceptibility to infection because immune defenses are dependent on cell replication and the production of proteins with biological activities like immunoglobulins, cytokines and acute phase proteins [30]. In the present paper we showed that protein deprivation affects splenic B cell number, but not their ability to produce Igs. In general, innate and cell-mediated immunity is more sensitive to malnutrition than humoral immunity [9]. More recent investigations also indicate a reduced Th2 activity and a decrease in antibody production during a diet restriction [5], [23]. In addition, Ishikawa et al. found differential cytokine (IFN-γ, IL4) production by splenic cells from dietary restricted animals when they were cultured with distinct stimuli as Con A, LPS or *Staphylococcus aureus* [23].

However, in our experimental model there were no differences in the immunoglobulin production when different stimuli were used. Then, the impairment of the humoral immune response in MN mice observed in our experimental model [11], [19], [24] was probably due to quantitative rather than qualitative changes in splenic B cells.

The antibody response against infectious agents depends on the correct orchestration of B cell generation and differentiation. When we investigated the effect of nutritional deprivation on B cell progenitors by flow cytometry, we observed a significant reduction in the total B lymphocyte number with a significant decrease of immature B cells, accompanied by an increase in the percentage of mature B cells, but not by changes in the number of mature B cells. These observations agree with the results obtained by Fock et al. [31] in MN animals, describing prominent lymphopenia with depletion in the lymphoid lineage and changes in cellular development. Our results are also supported by recent evidence showing that BM B cell development is altered by nutritional deprivation in parallel with decreased serum leptin concentrations [32]. We observed that, in addition to B cell compartment, malnutrition induces severe histological alterations in BM of MN mice. The decrease of hematopoietic space with an increase of extracellular matrix observed in MN mice could be responsible for the impairment of the hematopoietic microenvironment, which could affect the interactions between cells and cellular signaling [6]. Protein deprivation also generated a decrease in the number of total BM granulocytes and erythroblasts. These results agree with previous studies that showed BM atrophy with a reduced number of leukocytes, granulocytes and lymphocytes [6], [31], [33].

BM is the main hematopoietic organ of an adult organism, it maintains a close relationship with the immune system, and is able to respond to an emergency in immune cells such as infection. The fast and efficient recovery of the immunity is of great importance to reduce the risk of infection and mortality in malnutrition. When the repletion diets were administered, the recovery of hematopoietic spaces was observed. However, only the inclusion of *L. rhamnosus* or FGM in the repletion treatment was able to achieve the normalization of the cellular compartments in BM. Here, we demonstrated for the first time the effect of a probiotic in hematopoiesis after a malnutrition state. The addition of *L. rhamnosus* or FGM to the diet led myeloid and lymphoid cells to reach normal values and restored the marrow architecture.

The T cell populations present in murine BM have hardly been studied in detail previously, mainly because of their relative rarity. It has been described that hematopoiesis is maintained by activated BM CD4^+^ T cells [34]. Therefore, we directed our attention to the investigation of the influence of the treatments with immunobiotics on CD4^+^ cells and its relationship with the hematopoiesis. In contrast to the findings of Fock et al. [31], we observed that the nutritional deprivation induced an increase of CD3^+^CD4^+^ and CD3^+^CD4^−^ (probably CD8+) T cells in BM. That increase was accompanied by a decrease of the spleen T cells. Moreover, recent data have shown that normal hematopoiesis is really an antigen-induced state maintained by constant activation of BM CD4^+^ T cells. This T cell population includes a large number of recently stimulated cells in normal mice whose priming requires the presence of the cognate antigens [35]. It is possible that the increase of BM CD4^+^ T cells is a consequence of the stress induced by the BM hypoplasia in malnutrition in an attempt to restore the normal hematopoiesis. The decrease of spleen T cells would be a result of their mobilization to the BM. In the present paper, all the repletion diets normalized the BM T cells. Our results contribute to the understanding of the effect of probiotics on the recovery of immunity after a malnutrition state.

A remarkable finding of this work is that the beneficial properties of *L. rhamnosus* CRL1505 were preserved in the fermented dairy product containing the immunobiotic strain. Moreover, FGM was the only treatment able to normalize the absolute number of immature B220 cells after 7 days of repletion. Thus, it is likely that the effect of FGM depends not only on *L. rhamnosus* but also on non-bacterial components such as bioactive peptides [21].

At the moment, patients with immune deficiencies should probably only receive probiotics in the setting of a carefully conducted clinical research protocol. However, carefully selected and fully tested probiotic strains could provide additional options to improve the effect of conventional medical therapies. The present paper highlights the importance of immunobiotics in the recovery of the lymphopoyesis in immunocompromised hosts.

## Materials and Methods

### Microorganism

*Lactobacillus rhamnosus* CRL1505 was isolated from goat milk and kept at the CERELA culture collection. The LAB (stored at −70°C) was activated and cultured for 12 h at 37°C (final log phase) in Man-Rogosa-Sharpe (MRS) broth. The bacteria were harvested by centrifugation and washed three times with sterile 0.01 mol/l phosphate buffer saline, pH 7.2.

#### Fermented goats' milk

The FGM was prepared, using a starter culture specially designed for the fermentation of goat milk, as previously described [21]. The starter culture is constituted by *Streptococcus thermophilus* CRL806, *S. thermophilus* CRL728, and *L. rhamnosus* CRL1505 (strain with immunomodulatory properties), from the CERELA culture collection [20], [21]. The starter culture was inoculated at 1.25% (v/v) in pasteurized goat's milk and incubated at 42°C for 4 h. The FGM was stored at 4°C during the feeding period. The *L. rhamnosus* concentration was 6.2 log CFU/ml [21] in the final product.

### Animals and feeding procedures

Male 3-week-old Swiss albino mice were obtained from the closed colony kept at CERELA and they were housed individually in plastic cages at 25°C. Weaned mice were fed with a protein-free diet for 21 days, and the animals that weighed 45–50% less than well-nourished mice were selected for the MN mice experiments. Well-nourished control (WNC) mice consumed a balanced conventional diet (BCD) *ad libitum* [11]. MN mice were divided into different groups for repletion treatments: 1) BCD for 7 and 14 consecutive days (BCD groups); 2) BCD for 7 and 14 days with *L. rhamnosus* CRL1505 supplementation (10^8^ cells/mouse/day) [20] during the last 5 days of each treatment (BCD+Lr groups). 3) BCD for 7 and 14 days with FGM supplementation (that contains 10^6^ cells of *L. rhamnosus* CRL1505/g) during the last 5 days of treatment (BCD+FGM groups). *L. rhamnosus* and the FGM were administered *ad libitum* together with the BCD. The MNC group received only a protein-free diet while the WNC mice consumed the BCD *ad libitum*. All experiments were carried out in compliance with the Guide for Care and Use of Laboratory Animals and approved by the Ethical Committee of Animal Care at CERELA under the protocol BIOT-CRL/10.

### Cell preparation

Spleens from the different experimental groups were obtained and homogenized through a tissue strainer with RPMI 1640. BM cells were isolated by flushing femurs of mice with RPMI-1640. The red cells were lysed in Tris-ammonium chloride buffer (Sigma Aldrich) for 5 min at 4°C. Viable mononuclear cell numbers were determined by trypan blue exclusion using a Neubauer counting chamber.

### Flow cytometry studies

Spleen or BM cell suspensions were washed twice in ice-cold FACS buffer (Physiological solution, 2% fetal bovine serum, Gibco) and pre-incubated with anti-mouse CD32/CD16 monoclonal antibody (Fc block) for 30 min at 4°C. Cells were incubated in the antibody mixes for 30 min at 4°C and washed with FACS buffer. The following antibodies from BD PharMingen were used: FITC-labeled anti-mouse Mac-1, PE-labeled anti-mouse Gr-1, FITC-labeled anti-mouse CD3, PE-labeled anti-mouse CD4, FITC-labeled anti-mouse IgM, biotinylated anti-mouse B220 and biotinylated anti-mouse IgD antibodies or biotin conjugated antibody. In all cases, cells were then acquired on a FACSCanto II flow cytometer (BD Biosciences) and data were analyzed with FlowJo software (TreeStar).

### Total immunoglobulin determination

Splenic cells from WNC and MNC mice were cultured with medium alone, CpG-ODN 1826 (2 µg/ml, Operon Technologies) or LPS (10 µg/ml, Sigma) during 48 h. Total IgM and IgG concentrations were determined by ELISA as previously described [36], [37]. In brief, plates were coated with 2.5 µg/ml of the type-specific goat anti-mouse IgM or IgG (Sigma-Aldrich) overnight at 4°C, and blocked with 1% bovine serum albumin. Culture supernatants were incubated overnight at 4°C. Peroxidase-conjugated anti-mouse IgG or anti-mouse IgM (2.5 mg/ml) were added and incubated for 1 h at 37°C. The reaction was developed with TMB Substrate Reagent (BD OptEIA™). The concentration was measured with reference to standard curves using known amounts of the respective murine Ig (Sigma-Aldrich).

### Histological studies

The femoral from the different experimental groups was removed and immediately immersed in 4% paraformaldehyde, decalcified in 50% formic acid and 15% sodium citrate, and processed by standard histological techniques (paraffin-embedding). Sections measuring 5 µm from femur were stained by Haematoxylin-Eosin (HE), Alcian Blue pH 2.5 and Periodic Acid Schiff [6].

### Cytological studies

BM differential cell counts were carried out with smears stained with May Grünwald Giemsa under a light microscope (100×), and absolute numbers were calculated [14].

### Statistical Analysis

Experiments were performed in duplicate and results were expressed as mean values and standard deviations. A two-way ANOVA test was used. Tukey's test (for pair wise comparisons of the mean of the different groups) was used to test for differences between the groups. Differences were considered significant at p<0.05.

## Supporting Information

Table S1 BM B220 lymphocytes (10^5^ cells/femur).
(DOCX)
