# Supplementary material for: Dietary Supplementation with Probiotics Improves Hematopoiesis in Malnourished Mice
Source: PLoS One. 2012 Feb 8;7(2):e31171. doi: 10.1371/journal.pone.0031171 (PMC3275617; doi:10.1371/journal.pone.0031171)
Supplement: Table S1 — BM B220 lymphocytes (105 cells/femur). (DOCX) [file pone.0031171.s001.docx]

| **Groups** | **CD24+ IgD- cells** | **CD24+ IgD+cells** |
| --- | --- | --- |
| WNC | 59.11 ± 14.29ª | 5.35 ± 2.26^b^ |
| MNC | 14.37 ± 5.91^c^ | 4.62 ± 1.15^b^ |
| BCD7d | 41.01 ± 4.72^b^ | 0.75 ± 0.17^c^ |
| BCD+Lr7d | 45.44 ± 8.33^b^ | 0.34 ± 0.09^c^ |
| BCD+FGM7d | 51.35 ± 12.83ª | 0.37 ± 0.14^c^ |
| BCD14d | 65.71 ± 12.12ª | 8.14 ± 2.00^a^ |
| BCD+Lr14d | 68.80 ± 14.88ª | 7.25 ± 1.58ª |
| BCD+FGM14d | 64.23 ± 19.70ª | 8.09 ± 0.69ª |

**Table S1.** BM B220 lymphocytes (10^5^ cells/femur)

BCD: balanced conventional diet, BCD+Lr: BCD with supplemental *Lactobacillus rhamnosus*, BCD+FGM: BCD with supplemental fermented goat milk, WNC: well-nourished control, MNC: malnourished control.

Cells obtained from BM were stained with fluorochrome labeled anti-mouse B220, anti-mouse CD24 and anti-mouse IgD antibodies and analyzed by flow cytometry. The percentage of CD24 and IgD expressing B220 was expressed in absolute numbers.

^a,b,c^Mean values within a column with unlike superscript letters were significantly different (p<0.05).
